# Role of the HaHOG1 MAP Kinase in Response of the Conifer Root and But Rot Pathogen (Heterobasidion annosum) to Osmotic and Oxidative Stress

**DOI:** 10.1371/journal.pone.0031186

**Published:** 2012-02-01

**Authors:** Tommaso Raffaello, Susanna Keriö, Fred O. Asiegbu

**Affiliations:** 1 Department of Forest Sciences, University of Helsinki, Helsinki, Finland; 2 Viikki Graduate School in Molecular Biosciences (VGSB), Helsinki, Finland; Seoul National University, Republic of Korea

## Abstract

The basidiomycete *Heterobasidion annosum* (Fr.) Bref. *s.l.* is a filamentous white rot fungus, considered to be the most economically important pathogen of conifer trees. Despite the severity of the tree infection, very little is known about the molecular and biochemical aspects related to adaptation to abiotic stresses. In this study, the osmotic and oxidative tolerance as well as the role of the *HaHOG1* Mitogen Activated Protein Kinase (MAPK) gene were investigated. The transcript levels of the yeast orthologues *GPD1*, *HSP78*, *STL1*, *GRE2* and the ATPase pumps *ENA1*, *PMR1*, *PMC1* known to have an important role in osmotolerance were also quantified under salt osmotic conditions. The *HaHOG1* gene was used for a heterologous expression and functional study in the *Saccharomyces cerevisiae* Δ*hog1* strain. Moreover, the phosphorylation level of HaHog1p was studied under salt osmotic and oxidative stress. The result showed that *H. annosum* displayed a decreased growth when exposed to an increased concentration of osmotic and oxidative stressors. *GPD1*, *HSP78*, *STL1* and *GRE2* showed an induction already at 10 min after exposure to salt stress. Among the ATPase pumps studied, *PMC1* was highly induced when the fungus was exposed to 0.2 M CaCl_2_ for 60 min. The heterologous expression of the *HaHOG1* sequence in yeast confirmed that the gene is able to restore the osmotolerance and oxidative tolerance in the *S. cerevisiae hog1*Δ mutant strain. The HaHog1p was strongly phosphorylated in the presence of NaCl, KCl, H_2_O_2_ but not in the presence of CaCl_2_ and MgCl_2_. The GFP-HaHog1p fusion protein accumulated in the nuclei of the *S. cerevisiae hog1*Δ cells when exposed to high osmotic conditions but not under oxidative stress. These results provide the first insights about the response of *H. annosum* to osmotic and oxidative stress and elucidate the role of the *HaHOG1* gene in such conditions.

## Introduction

Living cells are able to respond as well as adapt to changes in the environmental conditions. A wide range of biotic and abiotic stresses often affect the fitness of a specific organism, by altering the metabolism and biology, thus compromising the development and the growth. Fungi are naturally exposed to a diverse range of environmental conditions which could differ depending on the fungal lifestyle and ecological niche. Among abiotic stresses, changes in the osmolarity and oxidative stress level can greatly affect the viability of the organism. Fungi possess several intracellular pathways that are able to respond to alterations in the osmolarity and oxidative conditions. Stress-activated protein kinases (SAPK) are mitogen activated protein kinases (MAPKs) which are phosphorylated in a variety of stress related conditions [1].

The MAPK core module is composed by three main MAPKs: a primary MAP kinase kinase kinase (MKKK), a MAP kinase kinase (MKK) and finally a MAP kinase (MAPK) [1]. MKKKs are activated either by MAPK kinase kinase kinase (MKKKK) mediated phosphorylation or by interacting with small GTP-binding proteins. When activated, MKKKs actively phosphorylate the second member MKK. Once activated, the MKK recognises and phosphorylates a conserved Thr-X-Tyr motif on the final MAPK effector. The MAPK can then specifically induce the expression of transcription factors or activate other protein kinases, phospholipases and cytoskeleton-associated proteins [1] [2]. Human and plant pathogenic fungi have evolved different ability to tolerate a wide range of osmotic and oxidative stressors. The HOG (High Osmolarity Glycerol response) is one of the most investigated stress related pathways which was first described in *Saccharomyces cerevisiae* [3].

Earlier studies have elucidated the two component mechanism of the HOG pathway in the budding yeast; a phosphorelay system which is composed of Sln1p-Ypd1p and Ssk1p and known to specifically activate a MAP kinase cascade composed by Ssk2/22p-Pbs2p and Hog1p [4]. When the osmotic pressure increases in the media, the Hog1p localizes in the nucleus and activates specific transcription factors to promote an initial response and adaptation [5]. In the budding yeast, oxidative stress caused by t-BOOH and H_2_O_2_ can also stimulate the phosphorylation of Hog1p mediated by Pbs2 in a similar manner to what happens during osmolarity condition [6]. Human pathogens have developed specific mechanisms to defend themselves especially from high oxidative conditions which they encounter during the colonization process. The MAPK Hog1p of the human pathogen *Candida albicans* is actively phosphorylated when the fungus is exposed to oxidative (10 mM H_2_O_2_) and osmolarity (1.4 M NaCl) stress conditions [7].The *Cryptococcus neoformans* serotype A and D *hog1*ΔA and *hog1*ΔD mutant strains show both high sensitivity to osmolarity condition (1 M and 1.5 M KCl). On the other hand, HOG1 ortholog gene has a different role in oxidative stress response in the two serotypes: while the *hog1*ΔA mutant is hypersensitive to 2 mM and 3 mM H_2_O_2_, the *hog1*ΔD is resistant [8]. In *Aspergillus nidulans* the sakA (HOG1 ortholog) MAPK is activated by phosphorylation in both osmotic (0.6 M KCl) and oxidative (0.3 mM H_2_O_2_) stresses even if ΔsakA mutant strain does not show any reduced growth in hyperosmotic medium compared to the wild type [9]. The hyphal growth in the plant pathogen *Fusarium proliferatum* ΔFphog1 mutants is reduced when the fungus is exposed to different stressors like H_2_O_2_, NaCl and the non-ionic sorbitol in a concentration dependent manner [10].

In the rice pathogen *Magnaporthe grisea*, the Δ*osm1* mutant shows an impaired growth when exposed to hyperosmotic conditions (0.4 M NaCl, sorbitol or KCl) and fails to accumulate arabitol compared to the wild type, thus showing a correlation between OSM1 activity and accumulation of suitable polyols to protect the fungus from the osmotic shock [11]. In the citrus necrotrophic fungus *Alternaria alternata*, the AaHOG1 mutants show enhanced sensitivity to oxidants like 7.5 mM tert-butyl-hydroxyperoxide, 30 mM H_2_O_2_, and 2 mM menadione, and high ionic osmotic conditions, like 1 M KCl and NaCl salts [12]. A copy of the *HOG1* homologue gene is present in the Dead Sea fungus *Eurotium herbariorum*. The *EhHOG1* gene from *E. herbariorum* is able to restore the normal osmotolerant phenotype when introduced in *S. cerevisiae Δhog1* mutant strain [13]. Moreover, complemented *S. cerevisiae Δhog1* strain with *EhHOG1* sequence shows a higher growth capacity in Li^+^ supplemented media compared to the *S. cerevisiae* wild type [13]. This result suggests that *EhHOG1* gene is probably part of those specific mechanisms that allow *E. herbariorum* to adapt to high Li+ content of the Dead Sea water [13].

Mutants with impaired growth in high osmolarity condition have been described in the model ascomycete fungus *Neurospora crassa* [14]. The *os-2* mutant strain is sensitive to the fungicide fludioxonil and the growth is inhibited in media supplemented with 4% NaCl. The *os-2* gene encodes a *S. cerevisiae* Hog1p homologue MAPK that confers resistance to hyperosmotic conditions and sensitivity to phenylpyrrole fungicides [14]. *HOG1* homologue gene has also been studied in the endophyte fungus *Epichloë festucae* [15]. The *E. festucae* sakA cDNA when introduced into the *Schizosaccharomyces pombe sty1* mutant strain is able to restore the stress sensitive defect when exposed to osmotic (KCl, sorbitol, NaCl) and oxidative (H_2_O_2_) stressors [15]. Like *N. crassa os-2* mutant strain, the *E. festucae* ΔsakA shows enhanced sensitivity to osmotic stress and resistance to the fungicide fludioxonil, but not increased sensitivity to oxidative stress [15].

Despite extensive research on signal transduction cascades and their role in fungal growth and pathogenicity, almost nothing has been done in this respect on the conifer pathogen *H. annosum*. *H. annosum* (Fr.) Bref. *sensu lato* species complex is the causative agent of root and butt rot disease and it is the most important pathogen of conifer trees in the northern hemisphere [16] [17]. Three intersterile species have been described in Europe, the P, S and F-types named according to their host species preferences (pine, spruce and fir respectively). The European P-type *Heterobasidion annosum* (Fr.) Bref. prefers trees of the genus *Pinus* as its host, but also infects many other conifer and broadleaved tree species. The European S-type *Heterobasidion parviporum* Niemelä & Korhonen, infects mainly Norway spruce (*Picea abies* (L.) Karsten) and seldom other tree species. Based on studies on the extent of decay caused by *H. parviporum* and *H. annosum* in 60 year old Norway spruce stems, *H. parviporum* shows higher specialization for spruce as a host [18]. The European F-type *Heterobasidion abietinum* Niemelä & Korhonen infects species of the genus *Abies* [16] [19]. The economical losses caused by *Heterobasidion* species in Europe are estimated to be around 800 million euro annually [17]. In Finland, *H. parviporum* and *H. annosum* cause severe damages in stands of Norway spruce (*P. abies*) and Scots pine (*Pinus sylvestris* L.).

In this study, the osmotolerance and oxidative tolerance of the tree pathogen *H. annosum* P-strain were characterized. We also described the role of the *HaHOG1* MAP kinase, the yeast *HOG1* homologues MAPK which has been demonstrated to be involved in osmotolerance and oxidative tolerance in many other fungi. This study also confirms the possibility to use a heterologous system to study the function of *H. annosum* genes thus overcoming the limitations imposed by the lack of an efficient DNA-transformation system.

## Materials and Methods

### Fungal strains and growth conditions

*H. annosum* P-type (isolate 03012 kindly provided by Kari Korhonen, METLA Finnish Forest Research Institute, Finland) was maintained in MEG agar plates (0.5% Malt extract, 0.5%Glucose and 2% agar) and grown at room temperature in laboratory conditions. Fungal culture experiments were all performed at room temperature in MEG agar plates or MEG liquid media. Transformed *Escherichia coli* for plasmid replication was grown in LB plates or LB liquid media at 37°C. The *S. cerevisiae* strains used in this study were the wild type BY4742 (Euroscarf acc. num. Y10000: MATα; his3Δ1; leu2Δ0; lys2Δ0; ura3Δ0) and the Δ*hog*1 mutant YLR113w (Euroscarf acc. num. Y12724: BY4742:MATα; his3Δ1; leu2Δ0; lys2Δ0; ura3Δ0; YLR113w::kanMX4). Both strains were maintained in yeast extract-peptone-dextrose (YPD) 2% agar plates at 30°C or grown in YPD liquid media under shaking at 28°C. YLR113w strain carrying the pYES2 plasmid was selected and maintained in synthetic defined (SD)-URA^−^ selective media.

### Gene cloning

The *H. annosum* P-type *HOG1* gene homolog was retrieved from JGI *Heterobasidion* genome browser (http://www.jgi.doe.gov/) with a BLASTp search using the *S. cerevisiae* Hog1p protein sequence from *Saccharomycete* Genome Database (SGD, http://www.yeastgenome.org/) as a query. Full-length gene sequence was PCR amplified from *H. annosum* P-type cDNA using specific primers (Table 1) and the following PCR program: 95°C 3 min, 30 cycles (95°C 1 min-57°C 30 sec-72°C 1 min), 72°C 10 min. *HaHOG1* full-length cDNA was purified and cloned into pGEMT-easy vector (Promega, Finland) according to manufacturer's instruction. The gene was sequenced and the sequence was submitted to GenBank database (accession number JN127357). *HaHOG1* cDNA was cloned into pYES2 vector (Invitrogen) by single restriction reaction with *Eco*RI (New England Biolabs) creating pYES2-HaHOG1 construct. Correct orientation was checked by restriction analysis using *Xho*I (New England Biolabs).

**Table 1:** List of primers used in this study.

Gene	Forward Primer	Reverse Primer
*HaHOG1* 1	ATGTCTTTCGTCAAGCTCAG	CTAGCTACAACAGGCC
*HaHOG1* 2	ATGTCTTTCGTCAAGCTCAG	TTCAGGCGGCCGCTCAGTCCGCATGCGGCGCGGA
*GFP* 2	CTCAGTAAGCTTATGGTGAGCAAGGGCGAGGA	GAGCTTGACGAAAGACATCTTGTACAGCTCGTCCA
*GPD1*	TGTCTCGGTGGTGTCCCTAT	ACCATGGCTGATGGAAGACT
*HSP78*	CCGGCATACTATGTCTCGTCT	TAGGGCCTTCGTCGAACA
*STL1*	CGTTTACCACGACCAGAGC	AAGCTCAAGCCATGTGCAG
*GRE2*	GCGTATCGTCGTTACCTCATC	CCTTCTCCTTTACCTCCTCGAT
*ENA1*	CCATTCAGTTCTGAGCGAAAGT	ACCCATTCAGCATGGACAC
*PMR1*	GACGCTTGGAAGCACTAATGT	GCGTCAACGATGAGGAATTT
*PMC1*	TCAAGACGCTCTTCAACGAC	GTCTTGCTGCCCACGAAC
*GAPDH*	ATCGTTGAGGGCTTGATGAG	GTGGACGAAGGGATGATGTT

1 : primers used for *HaHOG1* cloning.

2 : primers used to create GFP-HaHOG1 fusion gene. Restriction sites are underlined (see Materials and Methods).

### Effect of salts and H_2_O_2_ on *H. annosum* growth

To study the effect of different osmotic and oxidative conditions on *H. annosum*, the fungal growth was quantified on MEG 2% agar plates supplemented with either calcium chloride (CaCl_2_), potassium chloride (KCl), magnesium chloride (MgCl_2_), or sodium chloride (NaCl) at concentration ranging from 0.05 M to 0.5 M. For NaCl salt, 1 M concentration was also tested. For the oxidative conditions, hydrogen peroxide (H_2_O_2_) was added at final concentration ranging from 1 mM to 5 mM. The plates were sealed with parafilm and incubated at room temperature. The growth of the fungus was measured at regular intervals, and monitored for approximately 3 weeks post inoculation. Four replicates for each different concentrations were prepared and the colony radius was measured from three different directions from the center in each plate. The plates for the control contained MEG 2% agar without salt or oxidative stressors.

### *S. cerevisiae* transformation

*S. cerevisiae* Δ*hog*1 mutant strain (YLR113W) was transformed by electroporation either with the empty pYES2 vector (Δ*hog*1+pYES2) or with the pYES2-HaHOG1 vector (Δ*hog*1+pYES2-HaHOG1). Briefly: YLR113W mutant strain was grown in YPD media at 28°C until OD_600_ = 3. The cultures were centrifuged at 3000 rcf at 4°C for 5 min then washed twice with sterile MilliQ water and resuspended in 5 ml 1 M sorbitol and kept on ice. A further centrifugation at 2000 rcf at 4°C for 5 min was performed and finally cells were resuspended in 150 µl of 1 M sorbitol. Cell suspension (40 µl) of the *S. cerevisiae* were transferred in 0.2 cm path cuvette (Bio-Rad) and electroporated (BIO-RAD Gene-Pulser XCell, V = 1.5 KV, 25 µF, 200 Ohm). Electroporated cells were immediately resuspended in 1 ml of 1 M sorbitol and 200 µl were plated in SD-URA^−^ selective plates for colony selection. Plates were incubated at 30°C until colonies appeared.

### *S. cerevisiae* complementation experiment

The *S. cerevisiae* wild type strain was grown in 5 ml YPD media while the Δ*hog*1+pYES2 and the Δ*hog*1+pYES2-HaHOG1 mutant strain were grown in 5 ml SD-URA^−^ liquid media at 28°C over night. For the osmotolerance experiment, YPD 2% agar plates supplemented either with 2% glucose or 2% galactose+1% raffinose were prepared for each condition to be tested: 0.5 M and 1 M of NaCl, KCl, MgCl_2_, and CaCl_2_. For the oxidative tolerance experiment, YPD 2% agar plates supplemented with 2% galactose+1% raffinose were used with either 3 mM, 4 mM or 5 mM H_2_O_2_. The wild type, Δ*hog*1, Δ*hog*1+pYES2 and Δ*hog*1+pYES2-HaHOG1 strains were quantified with a hemocytometer and a dilution series (10^5^, 10^4^, 10^3^, 10^2^ cells) were spotted in a row for each strain. The plates were incubated at 30°C for 4–10 days to allow comparison between the wild type and the mutant strains.

### RNA extraction and cDNA synthesis

Total RNA was extracted from *H. annosum* P-type with a modified CTAB protocol [20]. Briefly, the mycelium was filtered from the culture using Miracloth (ChalBiochem), wrapped in aluminum foil, and immediately frozen in liquid nitrogen. For each sample, 3 ml CTAB extraction solution (2% (w/v), 100 mM Tris-Cl pH 8, 20 mM EDTA pH 8, 1,4 M NaCl, 2% (v/v) 2-mercaptoethanol) was added to the pulverized mycelium and the mixture was incubated for 30 min at 65°C. All samples were extracted twice with an equal volume of chloroform-isoamyl alcohol (24∶1) and centrifuged at 10000 rcf for 10 min at 20°C. Selective RNA precipitation was performed by adding 1/4 volume of 10 M LiCl. After overnight incubation at 4°C, samples were centrifuged for 30 min and the pellet was resuspended in 500 µl SSTE (1 M NaCl, 0.5% SDS, 10 mM Tris-Cl, 1 mM EDTA, pre-warmed at 65°C). A further extraction with an equal volume of chloroform-isoamyl alcohol (24∶1) was performed as described above. The upper phases were recovered, and total RNA were precipitated with two volumes of absolute ethanol overnight at −20°C. The samples were then centrifuged at 10000 rcf for 15 min at 20°C. The total RNA pellet was washed with 80% ethanol, left to dry for 30 min and then resuspended in 50 µl nuclease free water. RNA samples were stored at −80°C. Total RNA was retrotranscribed as follows: 1 µg total RNA was treated with DNase (Promega), incubated at 37°C for 30 min and final DNase inactivation at 65°C for 10 min. Random primers (0.1 µg, Fermentas) were added to the mixture and the reaction was incubated at 65°C for 5 min followed by a quick cool down on ice. The retrotranscription was performed with RevertAid Reverse Transcriptase (200 U, Fermentas) according to the manufacturer's instruction. The cDNA was then diluted 20 or 40 times to be used for the quantitative real-time PCR (qPCR) or used without dilution for semi-quantitative PCR.

### HaHOG1 phosphorylation experiment, total protein extraction and western blot

*H. annosum* was grown in liquid MEG media for 4 weeks at room temperature. The salt osmotic stress was induced by adding either NaCl, KCl, MgCl_2_ or CaCl_2_ at 0.5 M final concentration to the liquid fungal culture. The oxidative stress was induced by adding H_2_O_2_ at 5 mM final concentration. After each stress induction, the fungal mycelia was quickly harvested at 1, 3, 10, 30 and 60 min post salt addition and immediately frozen in liquid nitrogen. Total proteins were extracted with the following protocol: 200 to 400 µg fungal mycelia were homogenised with mortar and pestle in liquid nitrogen. Followed by addition of 700 µl lysis buffer [(0.5 mM sodium deoxycholate, 20 mM Tris-HCl pH 7.6, 10 mM NaCl) with 1× protease inhibitor cocktail (Proteoblock Protease Inhibitor Cocktail, Fermentas) and 1× phosphatase inhibitor cocktail (Halt Phosphatase Inhibitor Cocktail, Thermo Scientific)] to the homogenised mycelia and thoroughly mixed. Sample slurry was centrifuged at 6000 rcf for 10 min at 4°C. For each sample, 600 µl of the supernatant were recovered and stored at −80°C. Total proteins were quantified (Protein Assay, Bio-Rad) and 7 µg were loaded on 10% SDS polyacrylamide gel for protein separation. After overnight transfer on nitrocellulose membrane (Amersham Hybond ECL, Amersham), HaHog1p phosphorylation level was quantified using anti-phospho-p38 monoclonal antibody (phospho-p38 MAPK (Thr180/Tyr182) (3D7) Rabbit 9215, Cell Signaling Technology). Signal detection was performed using Immun-Blot Goat Anti-Rabbit IgG (H+L)-AP Assay Kit (Bio-Rad). Equal protein loading was checked by staining the membranes with PageBlue Protein Staining solution (Fermentas).

### Gene expression analysis by Quantitative real-time PCR (qPCR)

The *GPD1*, *HSP78*, *STL1*, *GRE2*, *ENA1*, *PMR1*, *PMC1* and *GAPDH* gene models in *H. annosum* were found by BLASTp search in the JGI *Heterobasidion* genome browser using the *S. cerevisiae* genes from the *Saccharomyces* Genome Database (SGD, http://www.yeastgenome.org/) as query. Internal primers for qPCR analysis were designed using the Universal ProbeLibrary Assay Design Center (Roche, http://www.roche.com). The primers used in this study are shown in Table 1. LightCycler 480 SYBR Green I Master (Roche) was used with 5.5 µl of the diluted cDNA sample in a 15 µl total reaction volume. The following cycles were used in the LightCycler® 480 Instrument II (384 wells plates, Roche): pre-incubation at 95°C for 5 min, denaturation 94°C for 10 sec (4.8°C/s), annealing at 59°C for 10 sec (2.5°C/s), extension at 72°C for 10 sec (4.8°C/s), 40 cycles of amplification and final extension at 72°C for 3 min. The Ct values were automatically calculated using the LIGHTCYCLER 480 software, the transcript levels were normalized against *GAPDH* expression and the fold change was calculated based on the control with the Pfaffl method [21].

### Gene expression analysis by Semi-Quantitative PCR

The *ENA1*, *PMR1* and *PMC1* gene expression was visualized on ethidium bromide agarose gel by semi-quantitative PCR. The three pumps and the internal reference *GAPDH* were amplified using the same primers listed in Table 1. The PCR reaction mixture was set as follows: 1 µl cDNA (undiluted, see above), 2.5 µl DreamTaq™ Green Buffer (Fermentas), 0.5 µl dNTPs (10 mM each, Fermentas), 1 µl forward primer, 1 µl reverse primer, 0.65 µl DreamTaq™ Green DNA Polymerase (Fermentas) and water to 25 µl total volume. For *ENA1*, *PMR1* and *PMC1* the PCR cycle was as follows: 95°C for 3 min, denaturation 95°C for 30 sec, annealing 59°C for 30 sec, extension 72°C for 30 sec, 26 cycles of amplification and final extension at 72°C for 10 min. For the internal reference gene *GAPDH* the same conditions were used but with only 20 amplification cycles due to the higher initial amount of transcript. To visualize the transcript level, 10 µl of the reaction mixture were then loaded on ethidium bromide agarose gel and photographed under UV light.

### GFP-HaHog1p fusion protein

The *HaHOG1* gene was fused to the GFP for subsequent subcellular localization. *HaHOG1* was amplified from pYES2-HaHOG1 vector using specific primers to include the *Not*I restriction site at the 3′ end of the gene (Table 1). The GFP was amplified using specific primers to include an overlapping region with the *HaHOG1* at the 3′ end and the *Hind*III restriction site at the 5′ end of the reporter gene (Table 1). The two genes were fused together by a “fusion PCR” using Phusion® High-Fidelity DNA Polymerase (Finnzymes) and the following PCR program: pre-incubation at 98°C for 30 sec, denaturation 98°C for 10 sec, annealing at 58°C for 20 sec, extension at 72°C for 1 min, 30 cycles of amplification and final extension at 72°C for 10 min.

The GFP-HaHOG1fusion fragment was purified from gel and double digested at both ends using *Hind*III and *Not*I restriction enzymes in a single reaction according to the manufacturer's instruction. The fragment was then ligated into the pYES2 vector restricted with the same two enzymes generating the pYES2-GFP-HaHOG1 construct. The construct was transformed into the *S. cerevisiae* YLR113W Δ*hog1* mutant strain using the same procedures as for the yeast complementation experiment (see above). The yeast cells were photographed using a fluorescent microscope (Leitz, Laborlux S) equipped with a digital camera (Olimpus, DP50-CU).

## Results

### Growth of *H. annosum* under salt osmotic and oxidative stress conditions

**Figure 1:**
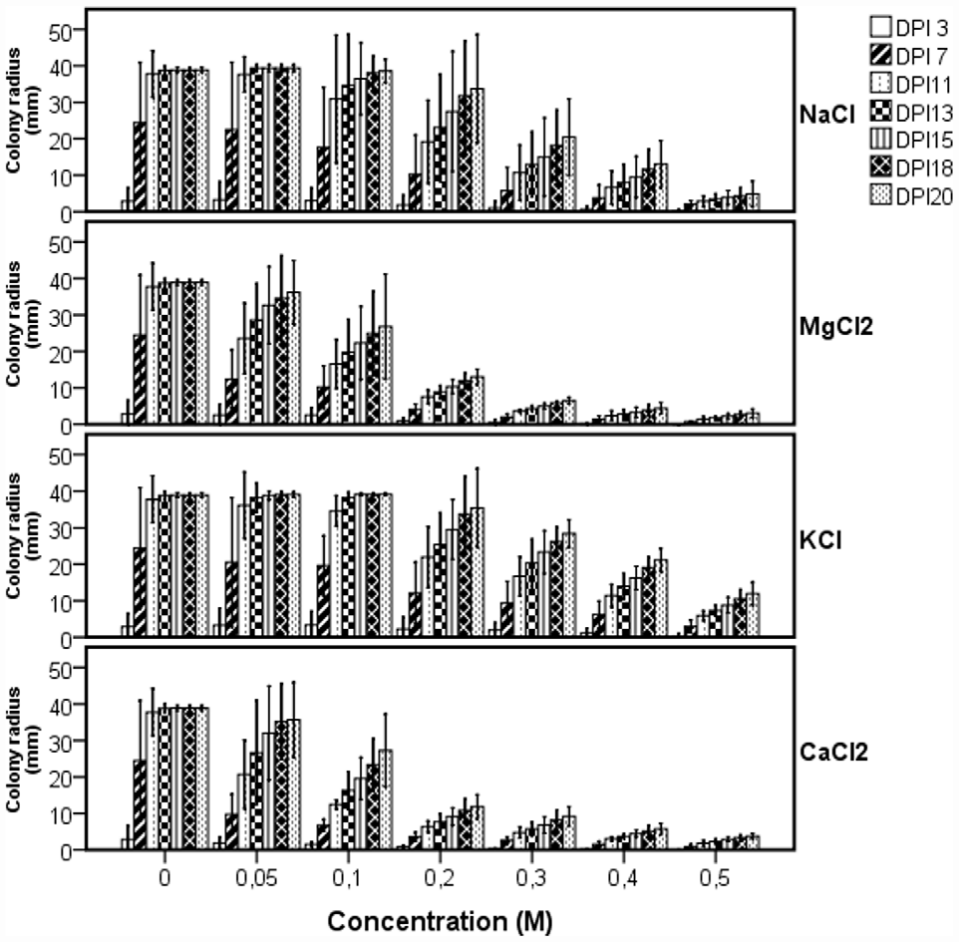
*Heterobasidion annosum* growth on MEG media supplemented with different salts at different concentrations. *H. annosum* colony radius was measured on MEG agar plate supplemented with NaCl, KCl, MgCl_2_ and CaCl_2_ at concentration ranging from 0 M to 0.5 M for 20 days. Radius was measured from 4 biological replicates (plates) and from 3 different directions in each plate. Bars represent standard deviation. DPI: Days Post Inoculation.

*H. annosum* displayed a decreased growth when exposed to osmolarity stress condition (Figure 1). The fungus can grow in media supplemented with NaCl, KCl, MgCl_2_ and CaCl_2_ with a concentration ranging from 0.1 M to 0.5 M. At 1 M concentration in all the tested salts, no growth was observed on the plates. The divalent salts (magnesium and calcium chloride) had a stronger inhibitory effect on the fungal growth compared to the monovalent salts (sodium and potassium chloride).

**Figure 2:**
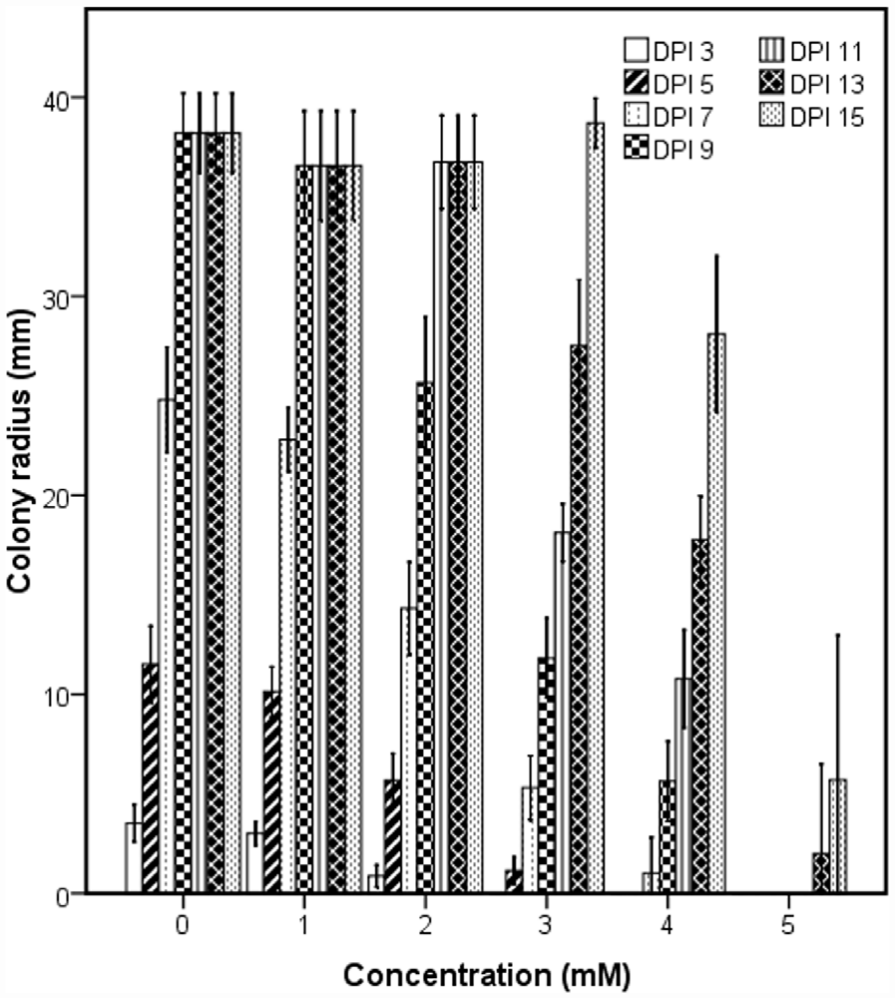
*Heterobasidion annosum* growth on MEG media supplemented with hydrogen peroxide at different concentrations. *H. annosum* colony radius was measured on MEG agar plate supplemented with H_2_O_2_ at concentration ranging from 0 mM to 5 mM for 15 days. Radius was measured from 4 biological replicates (plates) and from 3 different directions in each plate. Bars represent standard deviation. DPI: Days Post Inoculation.

*H. annosum* tolerated a concentration of hydrogen peroxide ranging from 1 mM to 5 mM (Figure 2). The fungal growth capacity decreased as the amount of H_2_O_2_ increased in the media. At 1 mM of hydrogen peroxide, the fungal growth was slightly inhibited compared to the control where the plate was covered by the fungal mycelium after 9 day post inoculation. At 3 mM H_2_O_2_ the fungal mycelium grew much slower and the petri-plate was over grown with hyphae at 15 days post inoculation (i.e. 6 days later than the control). At the highest concentration of 5 mM of hydrogen peroxide a much stronger inhibition was observed but the fungus was able to recover gradually with slow growth which started 12 days after initial inoculation.

### Gene expression under salt stress conditions

**Figure 3:**
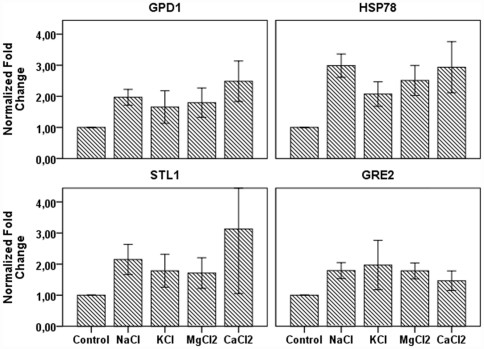
Expression of the genes *GPD1*, *HSP78*, *STL1* and *GRE2* in *Heterobasidion annosum* exposed to high concentration of different salts. The transcript levels of four putative *H. annosum* genes involved in the HaHOG1 osmotic pathway were quantified in the mycelium exposed to 0.5 M of either NaCl, KCl, MgCl_2_ or CaCl_2_ in liquid culture. RNA was extracted from the fungal mycelium, cDNA was synthesized and qPCR was performed. Fold change variation of the genes compared to the control was calculated using Pffafl method (*GAPDH* as internal reference was used). Three biological replicates were used for each treatment. Bars represent standard deviation.

**Figure 4:**
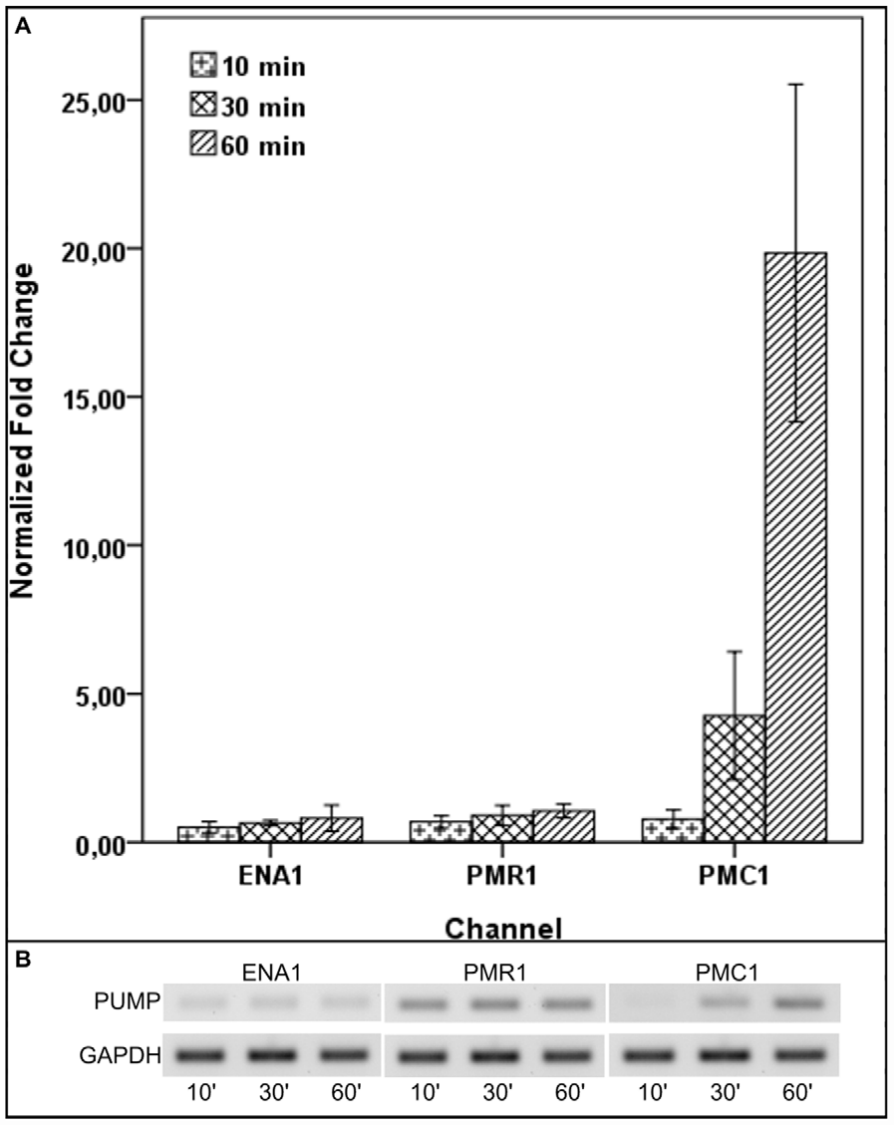
Expression of the ATPase pumps *ENA1*, *PMR1*, and *PMC1* in *Heterobasidion annosum* exposed to high concentration of calcium chloride. The transcript levels of three putative *H. annosum* ATPase pumps were quantified in the mycelium exposed to 0.2 M CaCl_2_ for 10, 30, 60 min in liquid culture. (A) RNA was extracted from the fungal mycelium, cDNA was synthesized, and qPCR was performed. Fold change variation of the genes compared to the control was calculated using Pffafl method (*GAPDH* as internal reference was used). Three biological replicates were used for each time point. Bars represent standard deviation. (B) A representative semi-quantitative PCR was performed on the three pumps using the same cDNA as for the qPCR. 10 µl of the PCR reaction mixture were equally loaded on ethidium bromide gel and photographed under UV light. Stable expression of the internal reference *GAPDH* transcript is shown.

The transcriptional regulation of 4 genes (*GPD1*, *HSP78*, *STL1* and *GRE2*) known to be associated with osmotic stress was investigated. The four genes were found up-regulated at 10 min after salt addition (NaCl, KCl, MgCl_2_ and CaCl_2_). The up-regulation was stronger for *HSP78* for which the level of the transcript was up to 3-fold induced compared to the control (Figure 3). *GPD1* showed a moderated up-regulation at 10 min after osmotic stress induction in all the conditions with an average induction of 2-fold compared to the control. The transcript level for *STL1* and *GRE2* was up-regulated compared to the control but not as strong as the other genes studied. The transcript level of three ABC transporters named ATPase *ENA1*, *PMR1* and *PMC1* were also quantified under different salt osmotic conditions by qPCR. When *H. annosum* was exposed to NaCl, KCl and MgCl_2_ the three pumps were expressed but no differences in the expression level was detected over time (data not shown). On the other hand, in the presence of the divalent salt CaCl_2_ neither *ENA1* nor *PMR1* showed any induction compared to the control although they were actively expressed to a certain level (Figure 4 A). However, the ATPase pump *PMC1* showed an increased expression at 60 min after the addition of CaCl_2_ to the *H. annosum* culture (Figure 4 A). The results were confirmed by the semi-quantitative PCR where an induction of *PMC1* over time in CaCl_2_ was also detected (Figure 4 B). In the semi-quantitative PCR gel picture, the pump transcript was almost absent after 10 min post calcium chloride addition but its transcript level increased after 30 min and 60 min (Figure 4 B). The *GAPDH* gene displayed a stable transcript expression thus providing a good reference gene for expression studies under salt conditions (Figure 4 B).

### *S. cerevisiae* complementation with *HaHOG1* under different salt conditions

**Figure 5:**
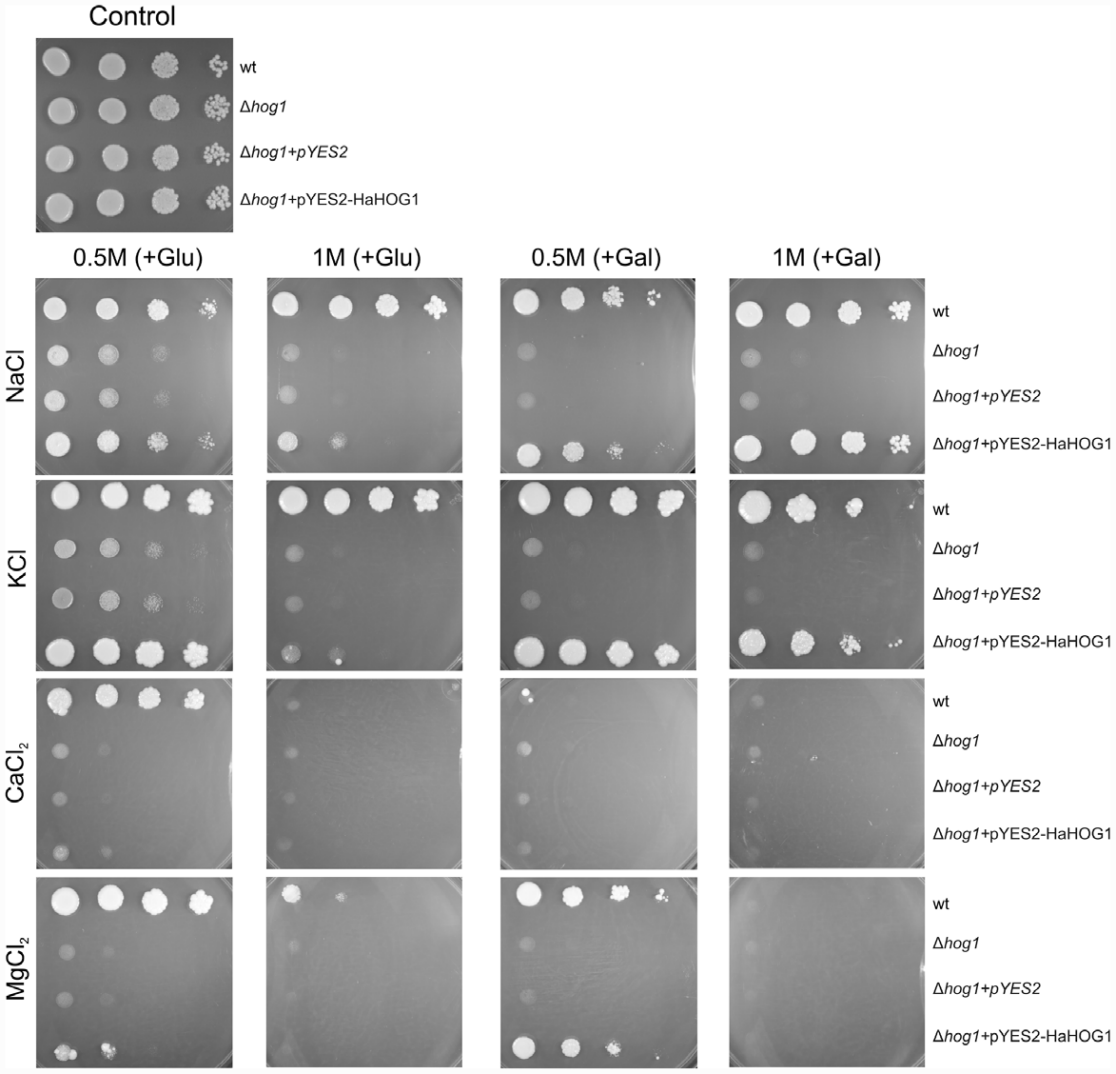
Complementation experiment using the *Heterobasidion annosum HaHOG1* gene in the *S. cerevisiae* Δ*hog1* mutant strain under salt osmotic conditions. The *H. annosum HaHOG1* gene was cloned into the pYES2 vector under the control of the galactose inducible GAL1 promoter and expressed in the osmosensitive *S. cerevisiae* Δ*hog1* mutant. Wild type and osmosensitive mutant were grown on YPD liquid media while the plasmid carrying yeasts were grown on selective SD-URA^−^ liquid media at 28°C. Four different salts were tested (NaCl, KCl, MgCl_2_ and CaCl_2_) with two different concentration (0.5 M and 1 M). For each salt two carbon sources were also used: either glucose (+Glu) to repress or galactose (+Gal) to induce the *HaHOG1* expression. Standard YPD media was used as control (Control). The four yeast strains used were as follow: wild type (wt), osmosensitive yeast strain (Δ*hog1*), osmosensitive yeast strain carrying the empty pYES2 plasmid (Δ*hog1*+pYES2) and osmosensitive yeast strain carrying the pYES2 plasmid with *HaHOG1* gene under the GAL1 promoter (Δ*hog1*+pYES2-HaHOG1). Cells from each strain were quantified with a hemocytometer, 10-fold diluted suspensions were prepare (10^6^, 10^5^, 10^4^, 10^3^ cells/ml) and 10 µl were spotted on the different plates. The plates were incubated at 30°C for 4–10 days to allow comparison between the wild type and the mutant strains.

A complementation experiment in *S. cerevisiae* Δ*hog1* mutant strain was performed with the *H. annosum HaHOG1* sequence. The result showed that the *H. annosum HaHOG1* gene is able to restore the capacity of the *S. cerevisiae* Δ*hog1* mutant strain to grow in high osmotic conditions (Figure 5). All the yeast strains were able to grow equally in standard YPD media supplemented with glucose (Control, Figure 5). The *S. cerevisiae* wild type strain (wt, Figure 5) is able to grow in media supplemented with 0.5 M and 1 M of sodium chloride (NaCl, Figure 5). In the same conditions, the Δ*hog1* mutants (Δ*hog1* and Δ*hog1*+pYES2, Figure 5) show a decreased growth, especially at the higher concentration of salts (1 M). However, the yeast Δ*hog1* mutant strain carrying the pYES2-HaHOG1 vector (Δ*hog1*+pYES2-HaHOG1, Figure 5) displayed the same osmotolerance compared to the wild type. The capacity to restore the osmotolerance is more evident when the expression of the *HaHOG1* gene in the pYES2 vector was induced in the presence of galactose in the media (+GAL, Figure 5). A partial complementation can be seen even under glucose repression condition when the yeast was exposed to 1 M of NaCl (+GLU, Figure 5). Probably a basal level of *HaHOG1* transcript was still present under the repression condition and this was enough to provide partial complementation in the mutant yeast. All the *S. cerevisiae* strains exposed to 0.5 M and 1 M of potassium chloride (KCl, Figure 5) displayed the same phenotype and osmotolerance as observed for the sodium chloride.

On the other hand, a stronger growth inhibition was observed in the presence of the divalent salts magnesium chloride (MgCl_2_, Figure 5) and calcium chloride (CaCl_2_, Figure 5) at the concentration of 0.5 M and 1 M. The wild type strain was able to grow at 0.5 M MgCl_2_ both in glucose and galactose media but it showed a remarkable reduced growth at higher concentration of salt (1 M) particularly in galactose condition. This result can be explained by the fact that galactose represents a poorer utilizable sugar compared to glucose. Furthermore, there were evidences related to the beneficial effect of the glucose regarding the osmotolerance of fungi (*C. albicans*) when exposed to a hyperosmotic condition [22]. The osmotolerance of Δ*hog1*+pYES2-HaHOG1 strain at 0.5 M MgCl_2_ is comparable to the wild type only when the *HaHOG1* gene was induced by the galactose (+GAL, Figure 5). The strongest toxicity effect among the salts is caused by CaCl_2_. Under calcium chloride osmotic condition, the only growth observed was related to the wild type strain at 0.5 M CaCl_2_ in the presence of glucose (+GLU, Figure 5).

### *S. cerevisiae* complementation with *HaHOG1* in oxidative conditions

**Figure 6:**
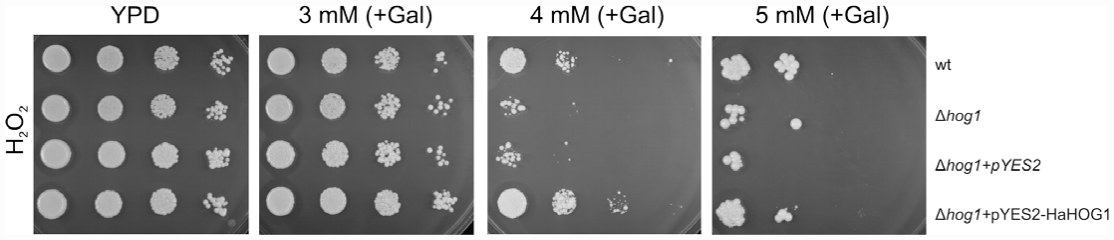
Complementation experiment using the *Heterobasidion annosum HaHOG1* gene expressed in the *S. cerevisiae* Δ*hog1* mutant strain under oxidative conditions. The *H. annosum HaHOG1* gene was cloned into the pYES2 vector controlled by the galactose inducible GAL1 promoter and expressed in the osmosensitive yeast Δ*hog1* mutant. Wild type and osmosensitive mutant were grown on YPD liquid media while the plasmid carrying yeasts were grown on selective SD-URA^−^ liquid media at 28°C. Different concentrations of hydrogen peroxide (H_2_O_2_) were used (3 mM, 4 mM and 5 mM). Galactose was used as carbon source to induce *HaHOG1* gene expression on the pYES2 vector. Standard YPD media was used as control (YPD). The four yeast strains used were as follow: wild type (wt), osmosensitive yeast strain (Δ*hog1*), osmosensitive yeast strain carrying the empty pYES2 plasmid (Δ*hog1*+pYES2) and osmosensitive yeast strain carrying the pYES2 plasmid with *HaHOG1* gene under the GAL1 promoter control (Δ*hog1*+pYES2-HaHOG1). Cells from each each strain were quantified with an hemocytometer, 10-fold diluted suspensions were prepare (10^6^, 10^5^, 10^4^, 10^3^ cells/ml) and 10 µl were spotted on the different plates. The plates were incubated at 30°C for 4–10 days to allow comparison between the wild type and the mutant strains.

The yeast strains were grown in YPD plate where the concentration of hydrogen peroxide ranged from 0.25 mM to 20 mM. A wide range of concentration was used to determine the sensitivity of *S. cerevisiae* strains to the oxidative stress. The results show that all the yeast strains (wt, Δ*hog1*, Δ*hog1*+pYES2 and Δ*hog1*+pYES2-HaHOG1, Figure 6) tolerated concentration of hydrogen peroxide up to 3 mM with no visible effect on the yeast cell survival. At the concentration of 4 mM H_2_O_2_ a remarkable difference in the yeast cell viability was seen between the mutant strains (Δ*hog1*, Δ*hog1*+pYES2, Figure 6) and the complemented strain (Δ*hog1*+pYES2-HaHOG1, Figure 6). Interestingly, the complemented strain shows a better fitness compared to the wild type strain when exposed to 0.4 mM of hydrogen peroxide. At 5 mM H_2_O_2_ the yeast cell viability was considerably decreased for all the yeast strains but still a better growth could be seen related to the complemented strain (Δ*hog1*+pYES2-HaHOG1, Figure 6) compared to the mutant strains (Δ*hog1*, Δ*hog1*+pYES2, Figure 6).

### HaHog1 phosphorylation level

**Figure 7:**
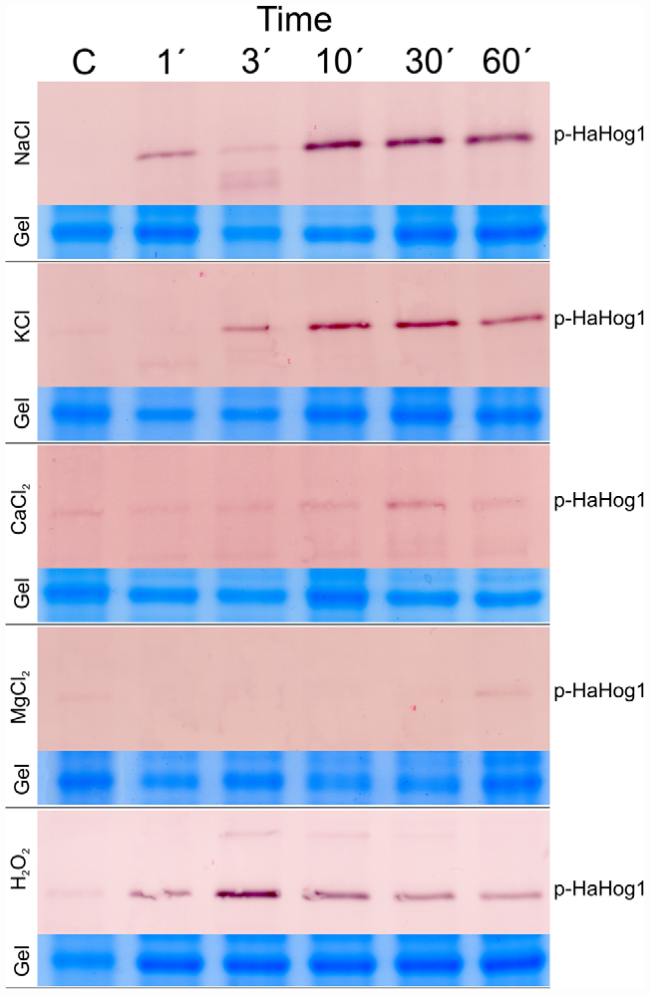
Phosphorylation level of the *Heterobasidion annosum* HaHog1p exposed to high concentration of different salts at different time points. The *H. annosum* mycelium was exposed to 0.5 M of NaCl, KCl, MgCl_2_ and CaCl_2_ in liquid culture and the total protein were extracted at 1, 3, 10, 30 and 60 min after the salt addition. 7 µg of total proteins were loaded on 10% SDS polyacrylamide gel for protein separation. The separated total proteins were transferred to a nitrocellulose membrane and the anti-phospho-p38 monoclonal antibody was used to detect the phosphorylated form of the HaHog1p.

The monoclonal antibody used in this study was able to reveal a sharp and specific band close to 46 KDa (Figure 7). The same antibody has been used in other studies and it showed cross-reaction among a broad range of species. Based on the literature data and the molecular weight which is close to the expected size (42.4 KDa, http://web.expasy.org/compute_pi/), we concluded that the band corresponds to the phosphorylated form of the *H. annosum* HaHog1p (phospho-HaHog1p). The western blot results showed an increased level of phospho-HaHog1p at 1 min and at 3 min after NaCl and KCl salt addition respectively compared to the control (Figure 7). The amount of phospho-HaHog1p increased gradually over time to reach the highest level between 10 min to 30 min in both salt conditions. However, in the presence of the divalent salts (CaCl_2_ and MgCl_2_) the amount of phospho-HaHog1p was considerably lower compared to the monovalent salts (NaCl and KCl) treatment (Figure 7). Indeed, a weak signal related to the phospho-HaHog1p appeared later at 30 min upon calcium salt addition and at 60 min upon magnesium salt addition.

In the case of H_2_O_2_ treatment, the phospho-HaHog1p signal was detected already after 1 min. The signal increased over time to reach a maximum at 3 min followed by a gradual decrease of signal intensity. However, a higher amount of phospho-HaHog1p compared to the control was still present at 60 min after hydrogen peroxide exposure (Figure 7).

### Subcellular localization of the GFP-HaHog1p in yeast

**Figure 8:**
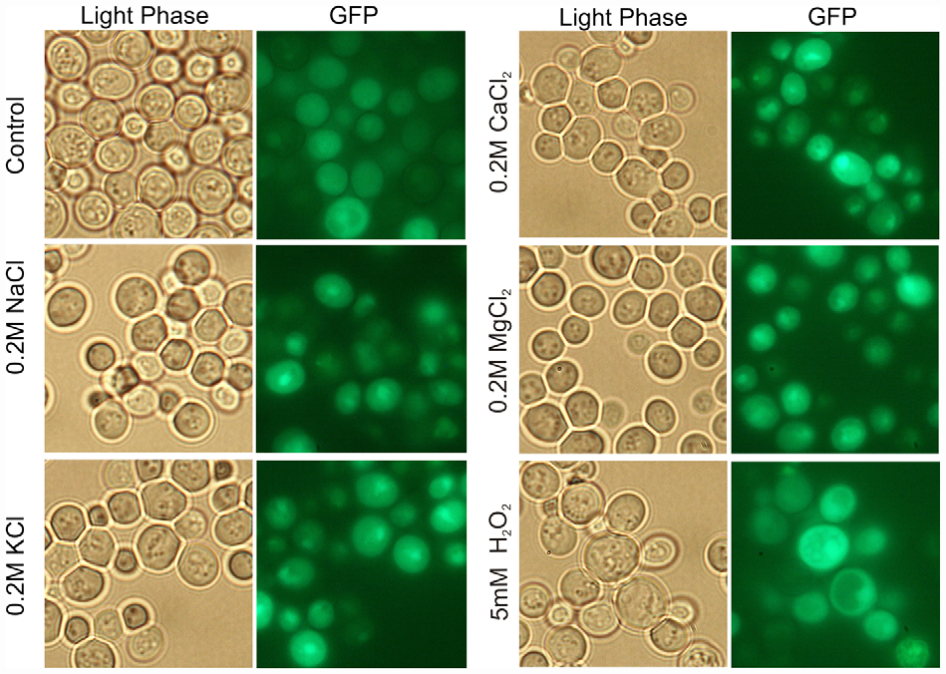
Subcellular localization of the GFP-HaHog1p fusion protein in *S. cerevisiae* Δ*hog1* mutant strain challenged with either osmotic stressors (O.2 M NaCl, KCl, MgCl_2_ and CaCl_2_) or oxidative stressor (5 mM H_2_O_2_). A GFP-HaHog1p fusion protein was generated and the subcellular localization was studied in the osmosensitive *S. cerevisiae* Δ*hog1* strain. The yeast cells show a cytosolic distribution of the GFP-HaHog1p protein in the absence of any stressors (Control). Accumulation of the GFP-HaHog1p protein in the nuclear compartment is seen as a brighter green spot in the cell when 0.2 M final concentration of different salts was added (NaCl, KCl, MgCl_2_ and CaCl_2_). No evident nuclear accumulation under oxidative stress condition in the presence of 5 mM of hydrogen peroxide (5 mM H_2_O_2_) can be seen in the condition used.

To study the subcellular localization of the HaHog1p protein in yeast, a GFP-HaHog1p fusion protein was generated. The yeast cells expressing GFP were observed in unstressed and stressed conditions under fluorescent microscope. The result revealed that the GFP fused to the N-terminal of the protein was still functional and the fusion protein localized in the cytoplasm in the unstressed yeast cells without any evident accumulation in any subcellular compartments (Control, Figure 8). The GFP in the N-terminal position did not alter the function of the *HaHOG1* gene since it can still restore the osmotolerance in high salt conditions when the construct was transformed into the *S*. *cerevisiae* Δ*hog1* mutant strain (data not shown). When the yeast cells were exposed to 0.2 M of the different salts, the GFP-HaHog1p accumulated in the nucleus of the stressed cell within the first 30 min. after the addition of the salt (NaCl, KCl, MgCl_2_ and CaCl_2_, Figure 8). The nuclear accumulation was confirmed by the co-localization with the DAPI staining (data not shown). Interestingly, no clear nuclear accumulation could be seen in the yeast cells exposed to 5 mM of H_2_O_2_ after 30 min following the addition of the oxidative stressor (Figure 8).

## Discussion

In this study, we investigated the response and the putative role of the MAPK *HaHOG1* in the basidiomycete *H. annosum* and its capacity to overcome osmolarity and oxidative stress conditions. A heterologous system with *S. cerevisiae* as a model host organism was used to study the function of the *HaHOG1* gene. The osmotolerance of *H. annosum* to osmotic stressors was investigated under different salt conditions. The results revealed that *H. annosum* was able to tolerate salt concentration less than 0.5 M. Tolerance capacity to higher salt concentration have been reported in other fungal species. *C. albicans*, *C. glabrata* and *Debaryomyces hansenii* show a higher tolerance level when exposed to NaCl osmotic stress compared to *H. annosum* [23]. In the plant pathogen *Botrytis cinerea* the wild type strain was able to grow in media supplemented with 1.5 M NaCl [24]. In another study, the wild type strain of the causative agent of Southern corn leaf blight, *Cochliobolus heterostrophus*, was able to tolerate concentrations of KCl up to 0.75 M [25]. In our study, monovalent salts (NaCl and KCl) inhibited the growth of *H. annosum* to a less extent compared to the divalent salts (CaCl_2_ and MgCl_2_). This result could be partly explained by the stronger osmotic pressure generated by divalent salts compared to the same concentration of the monovalent salts. On the other hand, Mg^2+^ and Ca^2+^ ions are known to have important intracellular roles in eukaryotes and are involved in critical intracellular function. In particular, Ca^2+^ ions act as a secondary messenger in pivotal intracellular pathways involved in cellular metabolism, growth and intracellular signal transduction [26]. In *Neurospora crassa* for instance, the role of calcium in the hyphal tip elongation has been described. Tip-high Ca^2+^ gradient and the role of actin cytoskeleton are pivotal for the site and direction of apical growth [27]. Very little studies have been carried out about the role of Mg^2+^ in fungal growth and development. Recently, in the wheat pathogen *F. graminearum*, it was shown that magnesium ions at low concentration (2 mM Mg^2+^) could inhibit almost completely the trichothecenes biosynthesis, an important class of mycotoxins [28]. Thus, high Ca^2+^ and Mg^2+^ ions in the media have the ability to affect the fungal growth not just by altering the cellular turgor but even by affecting intracellular pathways which are important for the fungal metabolism and biology. In a recent study, the levels of Na^+^, K^+^, Ca^2+^ and Mg^2+^ were quantified in the sapwood, heartwood, reaction zone and decay zone in Norway spruce infected by *H. annosum* [29]. It was shown that the above mentioned ions accumulated in the reaction zone (i.e. the area in the living wood with an active defense against the pathogen) as well as in the decay zone. In particular, K^+^ was detected at the highest amount in the decay area (4844 mg kg^−1^), followed by Ca^2+^ (1952 mg kg^−1^), Mg^2+^ (491 mg kg^−1^) and finally Na^+^ (20 mg kg^−1^) [29]. These data indicate that *H. annosum* encounters a high saline environment during its colonization process which suggests that it may have developed intracellular responses to be able to adapt in those unfavourable conditions.

No studies have been carried out about the capacity of *H. annosum* to tolerate high level of salts in the media. Consequently we investigated the expression of several putative genes which could be involved in osmotic stress tolerance according to literature data [30]. The NAD-dependent glycerol-3-phosphate dehydrogenase *GPD1* was shown to be an important induced gene during osmotic stress response [31].The *GPD1* induction in *H. annosum* after 10 min post salt addition was found to be close to 2-fold compared to the control. Our results correlates with the *GPD1* induction as it was described in *S. cerevisiae* 15 min after salt osmotic stress [32]. The *HSP78* gene encodes a mitochondrial heat shock protein and the transcript was found up-regulated in heat-shocked *S. cerevisiae* cells when grown in a non-fermentable carbon source [33]. It was found to be up regulated also in saline stress [30] and high hydrostatic pressure [34]. In *H*. *annosum* this is the first evidence of *HSP78* induction under salt stress with a transcript level as high as 3-fold compared to the level in the non-stressed mycelia. The transcript levels of two more genes, *STL1* and *GRE2*, were quantified. *STL1* encodes for a glycerol/H^+^ symporter in *S. cerevisiae* as demonstrated in a previous study [35]. In *C. albicans* the *STL1* expression was induced when the cells were exposed to osmotic shock (1 M NaCl) but a basal level of mRNA was still detected in the presence of either glucose or glycerol in minimal media (without stress) [36]. The major difference in the *STL1* expression from the non-pathogenic *S. cerevisiae* and the pathogenic *C. albicans* is that in the pathogenic fungus *STL1* is constitutively expressed in the presence of fermentable carbon source too [36]. In our study, the constitutive *STL1* expression in the presence of glucose could be the reason why we did not observe a strong induction of the putative *H. annosum STL1* transcript since the fungus was grown in media supplemented with glucose. Another reason could be that 10 min after salt addition is still too early to see a strong effect on *STL1* expression level caused by the osmotic stress. We also decided to quantify the expression level of the homologue of the *S. cerevisiae* methylglyoxal reductase *GRE2* [37]. As for *STL1*, we did not observe a strong *GRE2* transcript induction at 10 min post salt addition. In a previous microarray study, *GRE2* was shown to be induced in *S. cerevisiae* stressed cells at 10 min after osmotic shock imposition [38]. The discrepancy between literature data and our results could be explained by the different approaches used (microarray instead of quantitative PCR) or by the different organism studied.

The transcript levels of three different cellular channels (*ENA1*, *PMR1* and *PMC1* yeast orthologues in *H. annosum*) was also quantified by qPCR after exposure of the fungal cells to the different salts. No significant differences in the transcript level were found for *ENA1* and *PMR1* in the presence of NaCl, KCl and MgCl_2_ (data not shown). The yeast *ENA1* is an ATPase pump which is responsible for Na^+^/K^+^ efflux to keep the intracellular iron concentration at low level [39]. This type of pump is induced in the presence of high Na^+^ or K^+^ in the media in alkaline condition [40]. *H. annosum* was grown in acidified media (pH 5) and in low pH values it has been shown that other type of channels (electroneutral Na^+^/H^+^ and K^+^/H^+^ antiporters) are probably responsible for Na^+^/K^+^ cytosol depletion [40]. No induction at the transcript level was observed for the *PMR1* transcript either. The yeast *PMR1* is a Ca^2+^ ATPase pump responsible for the import of Ca^2+^ into the Golgi compartment and for the proper functioning of the secretory pathway [41]. Another pump, the yeast *PMC1* which is a vacuolar Ca^2+^ ATPase, is also responsible to keep the intracellular Ca^2+^ at a physiological state. Strong induction related to *PMC1* has been shown when the *PMR1* is not functional [42]. We observed a strong induction of *PMC1* homolog in *H. annosum* when exposed to 0.2 M CaCl_2_ for 60 min and this result provides the first evidence of the potential role of the *PMC1* pump in calcium homeostasis in this fungus. The level of *PMC1* transcript was very low in *H. annosum* in non-stress conditions and it was induced most probably to increase the sequestration in the vacuolar compartment of calcium thus keeping the intracellular concentration at an acceptable level (typically 0.1 µM). It should be emphasized that all the genes described in this study have not previously been functionally investigated. The results showed in this section were the first evidence about their possible role in the adaptation of *H. annosum* to osmotic stress. In future studies, the possibility to generate *H. annosum* knock-out mutants for the above mentioned genes will probably give more information about their precise role in the osmostress tolerance.

The sensitivity of *H. annosum* to oxidative stress was tested using hydrogen peroxide in the culture media. The fungus shows a decreased growth when the peroxide concentration increased with the highest inhibition at 5 mM. Similar to the osmotic stress, the sensitivity to oxidative stress varied between different fungi. *B. cinerea* can tolerate higher concentration of H_2_O_2_ up to 10 mM [24]. The plant pathogen *C. heterostrophus* can tolerate higher oxidative stress level with a range up to 20 mM of H_2_O_2_. The fungal lifestyle and its ecological niche could also have a profound effect on their response and tolerance to oxidative stress. The necrotrophic fungi *B. cinerea* and *C. heterostrophus* are actively and continuously exposed to the plant immune responses during the infection process. *H. annosum* is equally a necrotroph capable of killing living conifer tissues of all ages. It is also able to survive and adapt in heartwood tissues that contain many other toxic compounds. It would be of particular interest to investigate the ability of *H. annosum* to grow in the presence of toxic phenolic compounds which are abundant in the heartwood tissues. The differences in the resistance and susceptibility of *H. annosum* when compared to other fungal species would also provide some insights about the different oxidative tolerance ability based on host and infection process.

The activation of the *H. annosum* HaHog1p in osmotic and oxidative conditions was assessed by western blot using a monoclonal antibody able to recognize the phosphorylated form of the MAPK (phospho-HaHog1p). In the presence of the monovalent salts (NaCl and KCl), we observed the most strong and rapid HaHog1p activation within the first 60 min. A similar activation and kinetic pattern was also detected in other fungal species exposed to NaCl for the same period of time (0 to 60 min) [8] [43]. In this study, we showed that KCl, a less toxic salt compared to NaCl, can also activate the HaHOG1 pathway by phosphorylation of the HaHog1p MAPK. However, the sodium salt induced phosphorylation earlier at 1 min compared to the potassium salt for which the activation was induced at 3 min after stressor addition. A possible explanation could be the additive presence of the toxicity effect exerted by the Na^+^ ions compared to the K^+^ ions. Interestingly, both divalent salts, CaCl_2_ and MgCl_2_ at 0.5 M, were able to inhibit the HaHog1 phosphorylation. A very weak signal was recorded at 30 min for CaCl_2_ and at 60 min for MgCl_2_ after salt addition. In a recent study, the Hog1p activation was detected in *S. cerevisiae* exposed to 300 mM of CaCl_2_ [44] and the authors proposed a model for the activation of the HOG1 pathway by extracellular Ca^2+^ ions. In our study, we used a higher concentration of CaCl_2_ (0.5 M): such a high concentration of Ca^2+^ ions could have altered dramatically the cell physiology thus compromising the fungal stress adaptation pathways by inhibiting or delaying the HaHog1p phosphorylation. On the other hand, the impact of MgCl_2_ salt on the osmotic balance and on the physiology on the fungal cell has been less investigated. In our study, the effect of 0.5 M of MgCl_2_ was similar to that of CaCl_2_ causing an overall inhibition of the HaHog1p phosphorylation. The reason for such inhibition was not clear and this merits further investigation. The oxidative stress exerted by the use of hydrogen peroxide caused the HaHog1p to be clearly phosphorylated. The activation pattern in *H. annosum* is very similar to what was observed in *C. albicans* exposed to 10 mM of H_2_O_2_ [7]. In both fungi the phosphorylated Hog1p showed a peak around 2–10 min after oxidative stress exposure followed by a decrease in the signal intensity to a minimum at 60 min.

As no efficient DNA-transformation is available for *H. annosum*, a functional study of the *HaHOG1* gene was therefore carried out in the heterologous system *S. cerevisiae*. The high level of sequence similarity between *HaHOG1* and the yeast homolog *HOG1* (E-value = 1×10^−76^, and 68% of identity at the protein level) allowed the expression of the *H. annosum* sequence in the budding yeast despite the extreme diversity of the two organisms. The *HaHOG1* gene was able to functionally restore the osmotolerance in the *S. cerevisiae* Δ*hog1* mutant strain and the gene contribution to the osmotolerance was more evident when the *HaHOG1* gene was induced by the presence of galactose in the media. This suggests that *HaHOG1* could maintain the same functional role in the basidiomycete *H. annosum* by being part of the putative intracellular osmolarity pathway. All the orthologous MAPKs related to the core of the osmolarity pathway in *S. cerevisiae* have been annotated in the *H. annosum* genome thus reinforcing the hypothesis of a similar cellular response when the fungus was exposed to high salt condition. While the putative genes involved in osmotolerance are the same, the capacity to grow in high salt condition varies among different fungi. While *H. annosum* was not able to grow at 1 M NaCl, the yeast complemented with *HaHOG1* gene was able to tolerate this high salt concentration. This may partly be attributed to differences in the physiology of the two organisms. It is also possible that the factors regulating tolerance to high salt concentration does not reside in the *HaHOG1* gene itself, but much more in the overall fungal response that may differ in the upstream or downstream elements of the core osmolarity pathway. The type of carbon source in the media seems to have an impact in the osmotolerance of the fungus. In *C. albicans*, the presence of the glucose in the media has the effect to increase the fungal tolerance to KCl and NaCl [22]. In our case we observed a similar phenomena, the yeast wild type could grow better in the salt media supplemented with glucose compared to plates containing galactose.

Literature data suggests a possible pivotal role of the *HOG1* orthologue in oxidative stress condition. *Kluyveromyces marxianus kmhog1* mutant showed high sensitivity to 10 mM H_2_O_2_ compared to the wild type [45]. The *F. proliferatum Fphog1* mutant shows a remarkable decrease in the mycelia growth when exposed to 50 mM H_2_O_2_ compared to the wild type [10]. To test the involvement of the *HaHOG1* gene in such condition, we performed a yeast complementation test under different concentrations of hydrogen peroxide (H_2_O_2_) in plate supplemented with galactose to induce the *HaHOG1* expression. All the yeast strains (wt, Δ*hog1*, Δ*hog1*+pYES2 and Δ*hog1*+pYES2-HaHOG1) could grow equally well until the concentration of 3 mM H_2_O_2_. At this concentration of oxidant a better fitness can be seen in the Δ*hog1*+pYES2-HaHOG1 compared to the mutants and wild type. At the concentration of 4 mM of H_2_O_2_, a remarkable decrease in cell viability related to the Δ*hog1* was observed. Interestingly, the mutant strain complemented with *HaHOG1* sequence performed better than the mutant and the wild type itself. Under galactose conditions the *HaHOG1* is actively and strongly expressed by the presence of the inducible GAL1 promoter in the pYES2 vector. Because of that, the gene over expression could confer an advantage in terms of oxidative tolerance compared to the wild type. A comparison between the yeast and the *H. annosum* growth under oxidative stress conditions revealed a different behavior. While *H. annosum* showed a gradual decreased growth capacity which is concentration dependent in hydrogen peroxide in the range 1–5 mM, for *S. cerevisiae* no inhibition was observed until 4 mM when the growth was suddenly and considerably reduced.

To further prove the *HaHOG1* gene function in the heterologous system *S. cerevisiae*, we created a GFP-HaHog1p fusion protein. The activity of the *HaHOG1* gene fused to the GFP was tested with an independent complementation experiment: the pYES2-GFP-HaHOG1 construct was able to restore the osmotolerance in the Δ*hog1* yeast strain thus confirming that the *HaHOG1* function was not altered by the GFP. The GFP-HaHog1p localizes in the cytoplasm in unstimulated yeast cells and there was no clear evidence of any specific localization in any subcellular compartment. We observed that when the yeast cells were exposed to an increased osmolarity condition (simulated by adding 0.2 M NaCl final concentration) the GFP-HaHog1p strongly localized in the nuclei of the stressed cells within the first 30 min. The *S. cerevisiae* Hog1 MAPK was shown to cycle between the nuclei and the cytoplasm under non-stress conditions [46]. In the same study the authors showed that the Hog1p kinase strongly accumulates in the nucleus compartment in hyperosmotic conditions and later released into the cytoplasm upon stress adaptation [46]. The Hog1p was reported to localize in the nuclei even under oxidative stress conditions mediated by 1.5 mM tBOOH [47]. Recent studies showed that *HOG1* yeast ortholog in *A. alternata* accumulated in the nuclei when the fungus was exposed to H_2_O_2_ and fungicides [12]. In our study no clear nuclear accumulation under oxidative stress condition was observed. It is possible that the translocation occurs later compared to the osmotic stress in which the nuclear accumulation can be seen within the first 30 min. The discrepancy between the phosphorylation level of HaHog1p and the nuclear accumulation in *S. cerevisiae* cells could be explained by the different system in which the *H. annosum* MAPK was expressed. We cannot exclude that the conserved Hog1p could be regulated in a different way in the two biological systems. The possibility to investigate gene functions in this fungus is limited by the lack of an efficient transformation system and this poses major constraints to define the precise role of the single HOG1 pathway's components. Once an efficient DNA-transformation system is established, making single gene knock-out mutants will help to dissect the High Osmolarity Pathway in this fungus into its component to better understand its role in osmotic and oxidative stress. Finally, our findings provide the first insights about the response of the conifer pathogen *H. annosum* to osmotic and oxidative stress as well as elucidate the role of the *HaHOG1* gene in such conditions.
