# Correction: Role of the HaHOG1 MAP Kinase in Response of the Conifer Root and Butt Rot Pathogen (Heterobasidion annosum) to Osmotic and Oxidative Stress

**DOI:** 10.1371/annotation/f0434b66-c2a8-40c7-a488-44899cafcfb1

**Published:** 2013-07-30

**Authors:** Tommaso Raffaello, Susanna Keriö, Fred O. Asiegbu

The word "Butt" was spelled incorrectly in the article title. The correct title is: Role of the HaHOG1 MAP Kinase in Response of the Conifer Root and Butt Rot Pathogen (Heterobasidion annosum) to Osmotic and Oxidative Stress. 

The correct citation is: Raffaello T, Keriö S, Asiegbu FO (2012) Role of the HaHOG1 MAP Kinase in Response of the Conifer Root and Butt Rot Pathogen (Heterobasidion annosum) to Osmotic and Oxidative Stress. PLoS ONE 7(2): e31186. doi:10.1371/journal.pone.0031186. 

